# Biomechanical Evaluation of Different Posterior Mandibular Implant Configurations Under Bruxism: An FEA Study

**DOI:** 10.3390/jfb17080370

**Published:** 2026-07-31

**Authors:** Mehmet Ates, Ceyda Akin

**Affiliations:** Department of Prosthodontics, Faculty of Dentistry, Necmettin Erbakan University, 42090 Konya, Türkiye; ceydakin@hotmail.com

**Keywords:** finite element analysis, biomechanics, bruxism, zirconia, multi-unit, Ti-base, cantilever

## Abstract

This study evaluated the biomechanical performance of different abutment types, superstructure materials, prosthetic designs, and loading conditions in the implant-supported rehabilitation of three consecutive missing teeth in the mandibular posterior region using three-dimensional finite element analysis (3D-FEA). Twenty FEA models were constructed, simulating two-implant (pontic, mesial, and distal cantilever) and three-implant (splinted and unsplinted) configurations using multi-unit and Ti-base abutments composed of titanium alloy (Grade 5, Ti-6Al-4V), with monolithic zirconia and zirconia-supported feldspathic porcelain superstructures. Functional and parafunctional (bruxism) vertical and oblique loads were applied to analyze von Mises stresses in the components and principal stresses in the peri-implant bone. The results indicated that two-implant models generated higher stress concentrations than three-implant models, and unsplinted or cantilevered designs produced elevated stresses compared to splinted or pontic designs. Ti-base abutments resulted in greater stress accumulation in the connection complex compared to multi-unit systems. Under vertical and oblique parafunctional forces, stress values on the abutments in distal cantilever models—particularly in unsplinted Ti-base designs under oblique loading—exceeded the yield strength of the Ti-6Al-4V alloy (890 MPa), indicating a substantial risk of plastic deformation. To optimize biomechanical stability, three-implant-supported, splinted designs utilizing Multi-unit abutments should be prioritized. Avoiding distal cantilevers and unsplinted designs is critical in patients with bruxism to minimize the risk of mechanical failure in the Ti-6Al-4V components.

## 1. Introduction

Implant-supported fixed restorations have become the standard of care in contemporary dentistry, offering a highly predictable and reliable treatment option for the rehabilitation of missing teeth [1]. However, the long-term clinical success of implant therapy relies not only on achieving surgical osseointegration but also on the biomechanical balance that dictates how occlusal loads are transmitted to the implant, prosthetic components, and surrounding bone. The posterior mandible represents one of the most challenging areas for biomechanical stability due to its high masticatory forces, the prevalence of oblique loading, the proximity to the inferior alveolar nerve, and anatomical limitations regarding bone volume [2,3]. Unlike natural teeth, dental implants lack a periodontal ligament; consequently, occlusal forces are transmitted directly to the implant, abutment, and surrounding bone. This lack of shock absorption leads to crestal stress concentrations, which are recognized as primary triggers for marginal bone loss and mechanical failure [4,5].

Clinical decision-making for the rehabilitation of consecutive tooth loss in this region necessitates a multi-parametric evaluation, including implant number, splinting strategy, cantilever presence, abutment design, and restorative material selection. While increasing the number of implants may facilitate a broader distribution of occlusal forces, non-ideal positioning and distal cantilever extensions create a leverage effect, potentially leading to stress accumulation in the implant–abutment complex and peri-implant bone [6,7]. The degree of splinting further influences this stress distribution; while splinted superstructures promote a more homogeneous load distribution, unsplinted restorations are often preferred for passive fit and improved hygiene access [8]. Furthermore, connection stability is directly linked to the chosen abutment system. Multi-unit abutments, which facilitate a passive fit at the tissue level, are often compared with hybrid Ti-base systems that synthesize the mechanical strength of titanium with the esthetic advantages of CAD/CAM materials [9]. Variations in the elastic modulus of the crown materials used on these systems (e.g., monolithic zirconia vs. layered porcelain) fundamentally alter how stress is damped and transmitted to the bone [10].

These prosthetic considerations become exceptionally critical in the presence of bruxism, defined as repetitive jaw-muscle activity [11]. Increased occlusal forces impose additional loads on the implant–abutment interface, compromise screw stability, and challenge restorative material strength. Three-dimensional finite element analysis (FEA) serves as an indispensable research tool for investigating these complex stress distributions that cannot be directly assessed clinically, guiding decision-making by elucidating biomechanical trends under risk-prone loading conditions [12].

From a biomaterials perspective, understanding how connection components (e.g., Titanium Alloy Grade 5, Ti-6Al-4V) and restorative materials (e.g., monolithic zirconia ceramics) distribute stresses and resist mechanical fatigue under physiological and extreme parafunctional loading is crucial for optimizing the long-term clinical success of implant-supported complexes. Therefore, the primary objective of this research was to conduct a comparative biomechanical analysis—using 3D finite element analysis (3D-FEA)—to evaluate the stress distribution and structural response of various treatment configurations. Specifically, the study investigates the impact of implant numbers (two vs. three), cantilever presence, abutment systems (multi-unit vs. Ti-base), splinting strategies, and superstructure materials under both functional and parafunctional (bruxism) loading. The research tests the premise that variations in implant positioning, abutment design, and restorative material properties significantly modulate stress concentrations and plastic deformation risks within the peri-implant bone and prosthetic interface.

## 2. Materials and Methods

**Figure 1:**
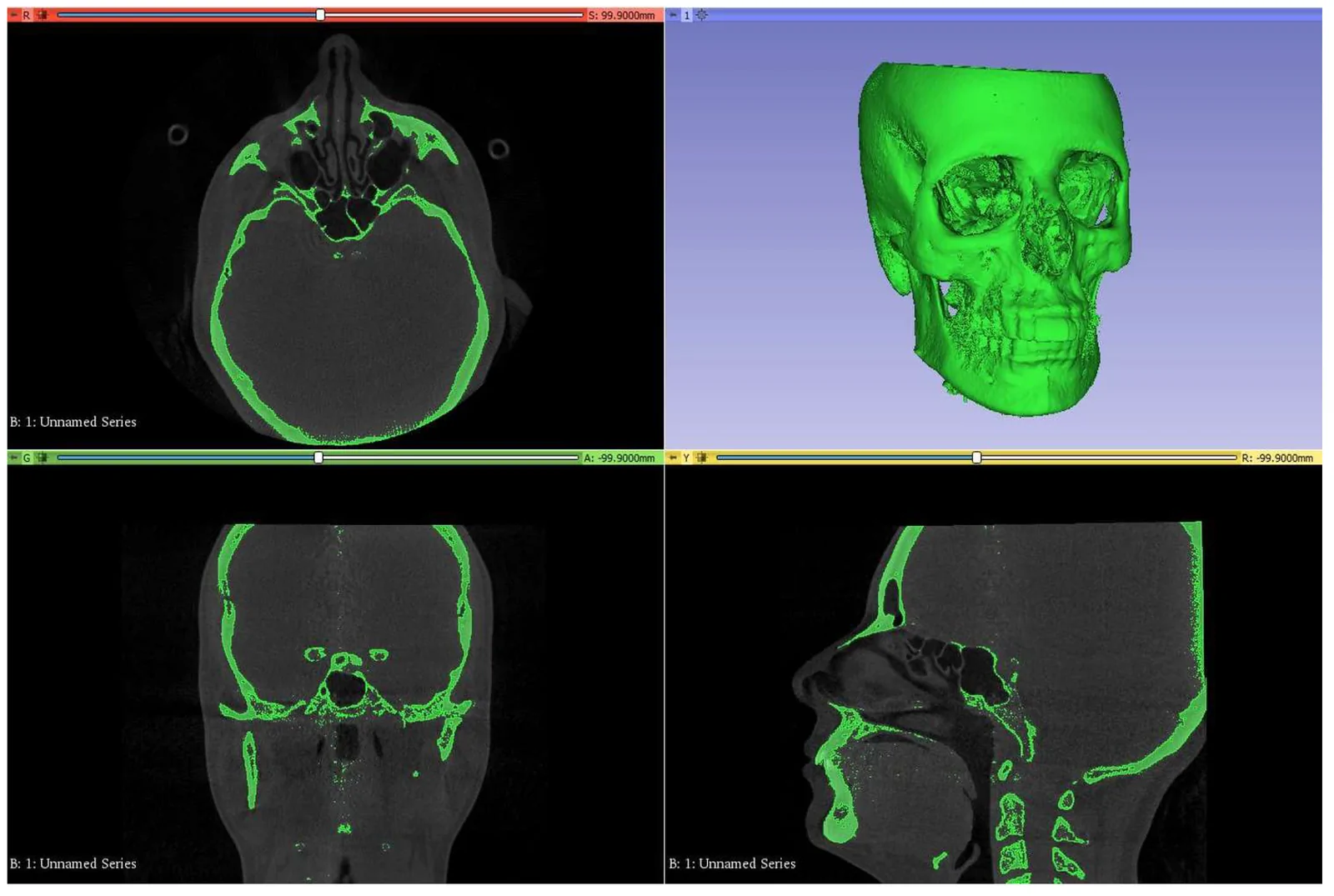
Generation of 3D anatomical models from the Visible Human Project CT dataset.

For this in silico study, the mandibular bone model was developed in collaboration with Tinus Technologies based on anatomical data retrieved from the Visible Human Project (National Library of Medicine, Bethesda, MD, USA). The geometric reconstruction was performed utilizing the high-resolution computed tomography (CT) dataset with a slice thickness of 0.33 mm obtained from this project (Figure 1). The DICOM images were segmented using 3D Slicer software An inward offset of 2 mm was applied to the anatomical model using Blender to delineate the cortical and trabecular bone layers. For the purpose of biomechanical standardization, the mandibular canal was excluded from the model [13].

**Figure 2:**
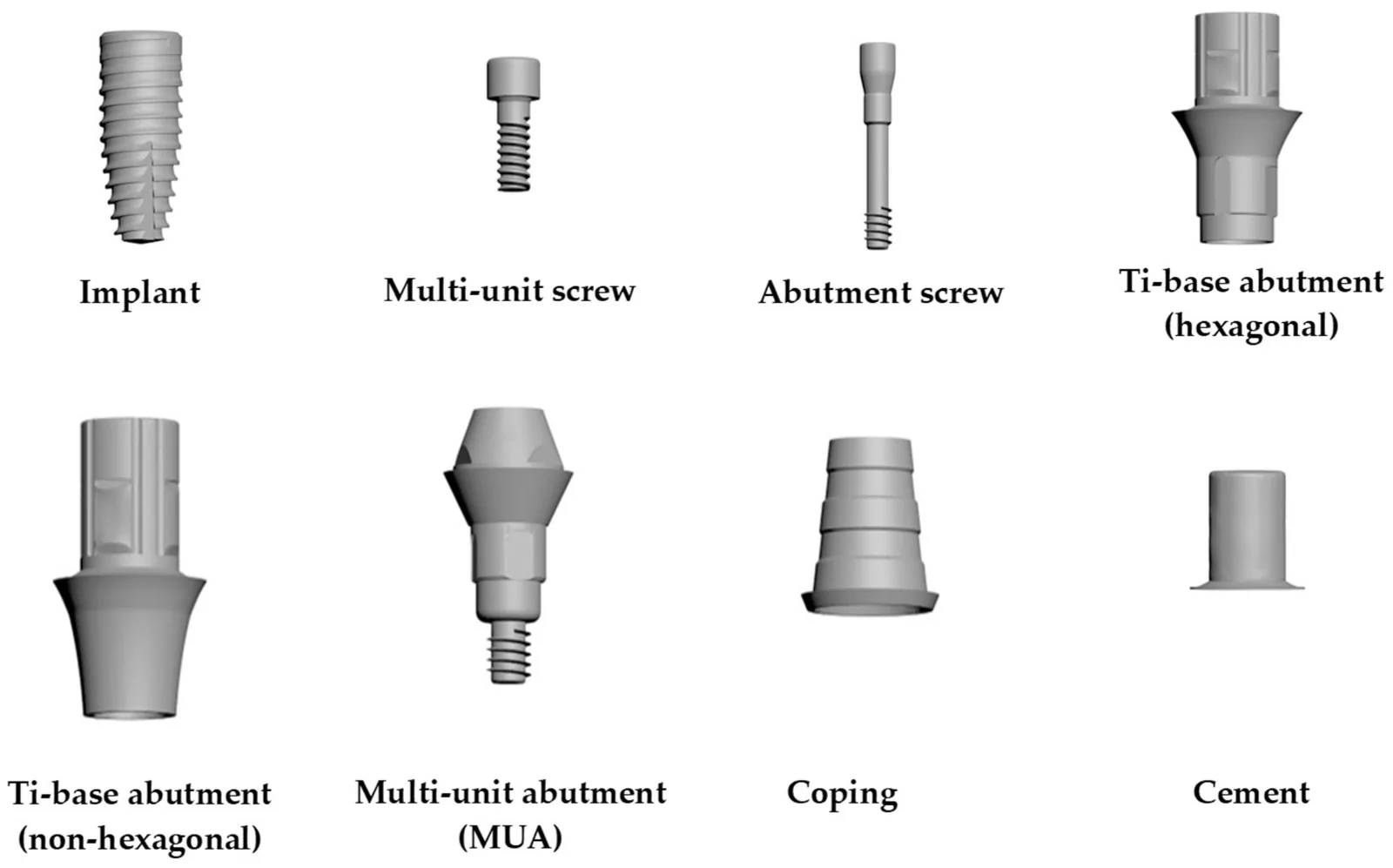
Models of the implant and prosthetic components.

Bone-level dental implants (4.1 × 10 mm, Straumann BLT, Basel, Switzerland), along with multi-unit and Ti-base abutments (1 mm gingival height), were utilized (Figure 2). The original industrial CAD data of these components were converted into solid models using SolidWorks (Dassault Systèmes, Waltham, MA, USA)) and integrated with the prosthetic crowns and bone anatomy. Reverse engineering processes were completed using Rhinoceros 3D (Robert McNeel & Associates, Seattle, WA, USA).

**Figure 3:**
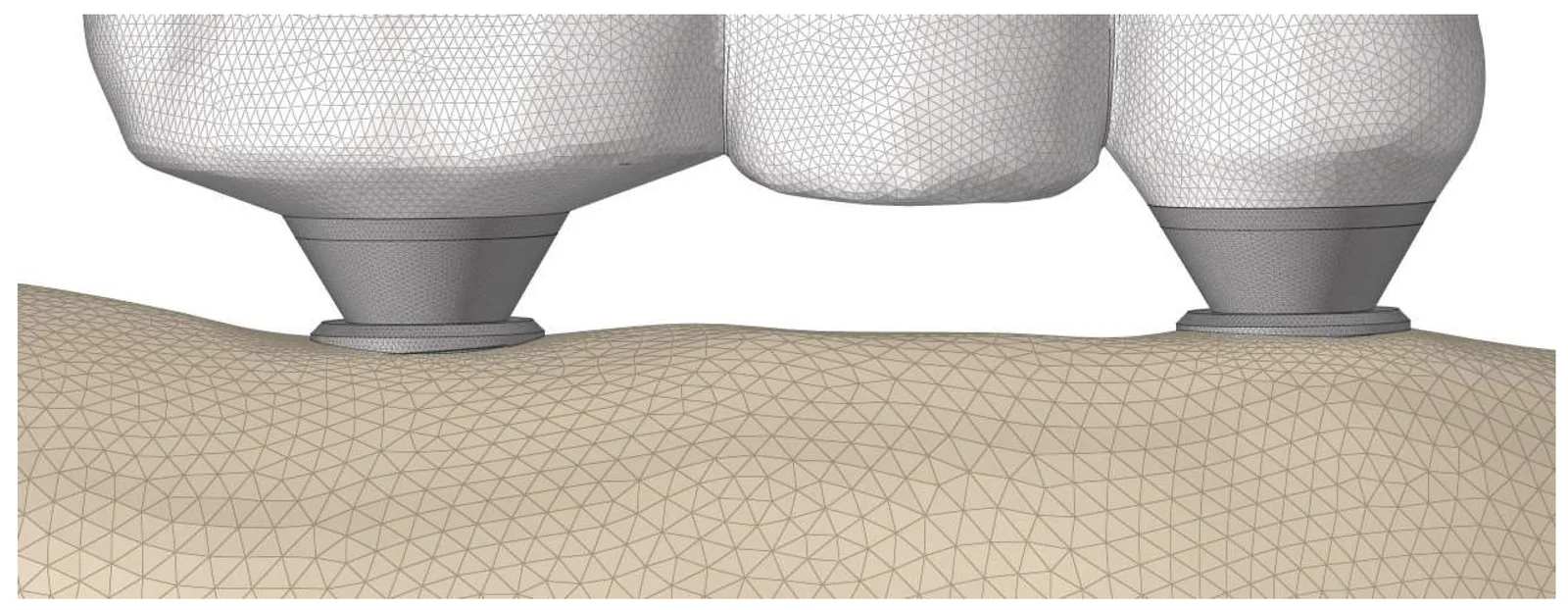
Models prepared in ALTAIR Hypermesh software (ALTAIR, Troy, MI, USA).

Finite element analysis (FEA) preparation, including mesh generation and interfacial adaptations, was performed using ALTAIR Hypermesh, while stress analysis was executed via ALTAIR Optistruct (ALTAIR, Troy, MI, USA). The geometry was discretized into finite elements; a mesh convergence study was conducted to ensure result accuracy. Anatomical surfaces were first discretized using triangular elements (0.1–0.5 mm), followed by volumetric meshing with 10-node tetrahedral solid elements. The resulting models ranged from 282,185 to 498,407 nodes and 1,103,010 to 1,978,906 elements. Localized mesh refinement (0.1 mm) was applied to critical areas—specifically at the implant–abutment interfaces and surrounding cortical bone—to capture steep stress gradients, while a global mesh size of 0.5 mm was maintained (Figure 3).

**Figure 4:**
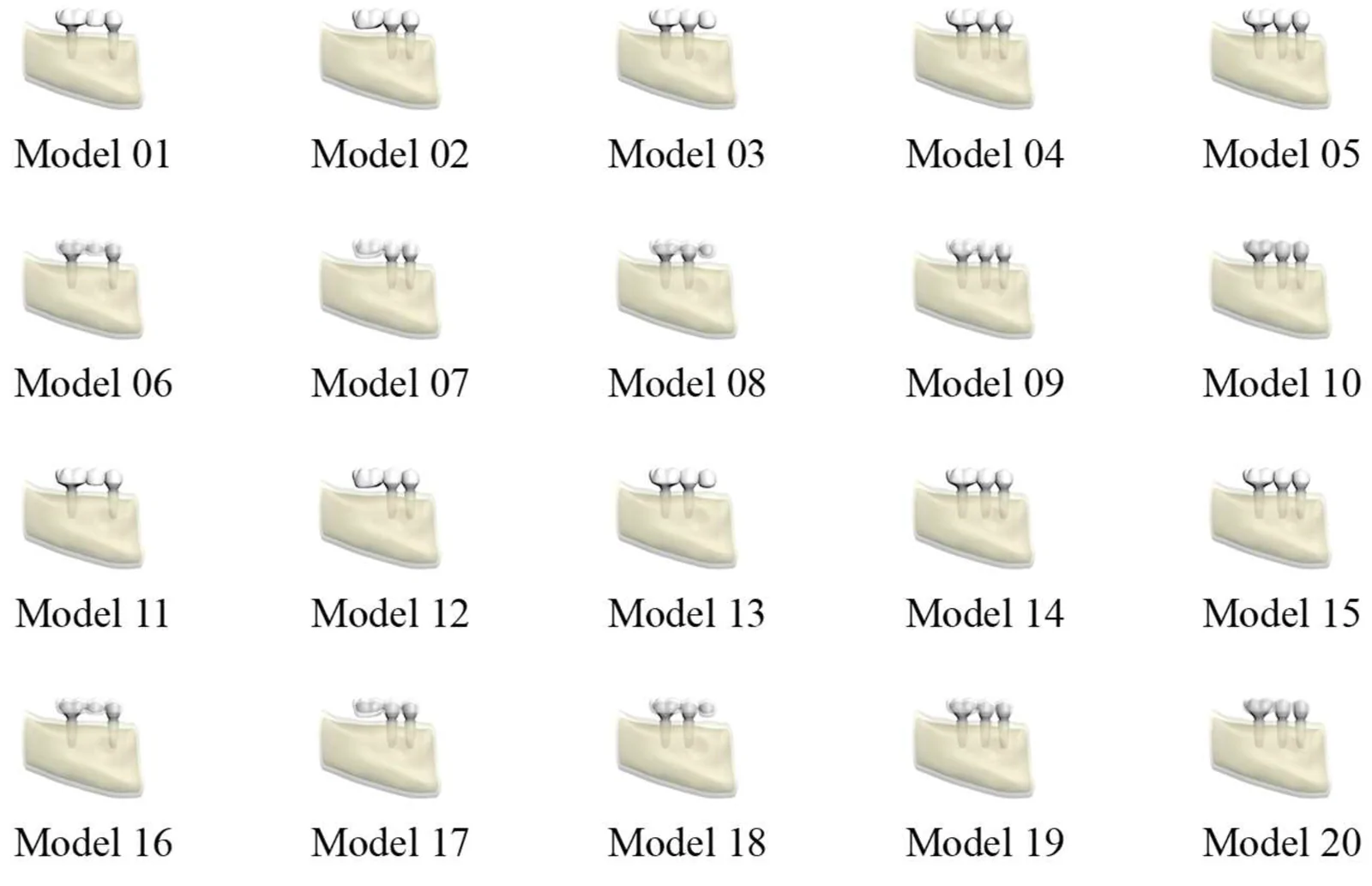
Images of all study models.

The missing teeth in the posterior mandibular region (teeth 44, 45, and 46) were modeled referencing Wheeler’s Dental Anatomy atlas [14]. Based on the implant positions, splinted, unsplinted, pontic, and cantilevered superstructure designs were generated. To ensure standardization, the total thicknesses of the monolithic zirconia restorations and the zirconia-supported feldspathic porcelain restorations were kept equal. To simulate clinical conditions more realistically, a 50-µm-thick dual-cure resin cement layer was defined between the abutment and the superstructure [15]. To evaluate the macro-mechanical load distribution without the confounding variables of micromotion, all structural interfaces within the system—including the bone–implant, implant–abutment, abutment–screw, and cement–crown boundaries—were mathematically modeled as perfectly bonded (FREEZE contact condition), assuming 100% osseointegration and absolute component rigidity. The grouping and visual representations of the 20 different models tested in this study are provided in Table 1 and Figure 4.

All structures used in the analysis were assumed to be linear elastic, homogeneous, and isotropic. The elastic modulus and Poisson’s ratio values for the materials were obtained from the literature and defined in the system (Table 2).

**Figure 5:**
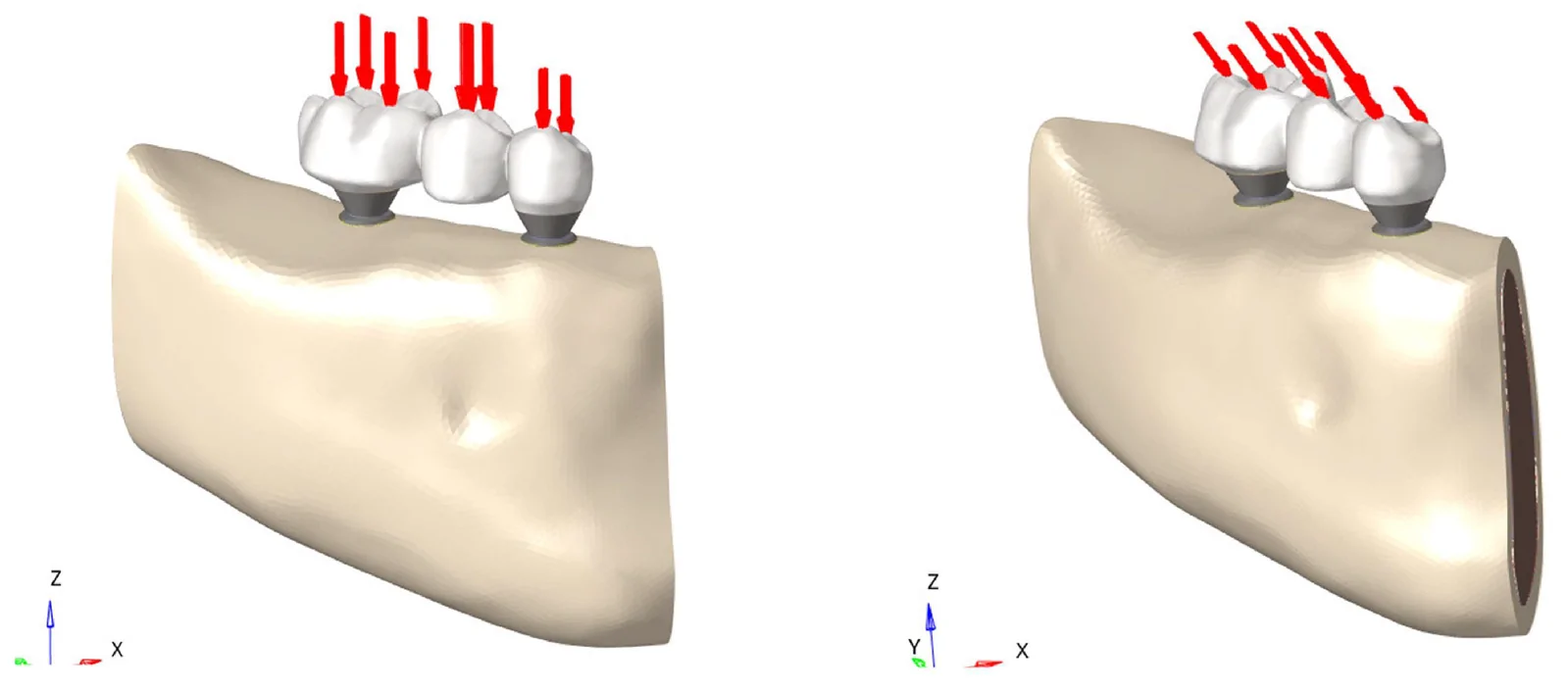
Schematic illustration of the boundary conditions, contact definitions, and static load applications (vertical: **left**; oblique: **right**) on the prosthetic restorations. The red arrows indicate the direction and application points of the simulated loads.

To simulate physiological and parafunctional (bruxism) masticatory forces, four different static loading scenarios were defined for each of the 20 models, resulting in a total of 80 analyses. To prevent stress singularity, the forces were applied by distributing them across the surrounding nodes in the loading areas. The loading scenarios and force distributions used in the biomechanical analyses are summarized in Table 3. Furthermore, Figure 5 serves as the schematic diagram illustrating the boundary conditions, load application points, and contact definitions to ensure full model transparency and reproducibility.

The loading magnitudes and directions were meticulously selected based on the established biomechanical literature. To simulate extreme parafunctional (bruxism) episodes, a 1000 N vertical load and a 500 N oblique load were applied, consistent with the protocols of previous studies [20,21,22]. The 30° angle for oblique loads was chosen to represent the transverse forces generated during lateral mandibular excursions, as validated in the literature [23]. Furthermore, deviating from traditional FEA studies that distribute total occlusal loads equally across all supports, this study adopted an anatomically accurate “proportional loading” strategy to maximize clinical reliability. Specifically, the total load was distributed as 50% to the first molar, 30% to the second premolar, and 20% to the first premolar. This ratio is justified by the mandible functioning as a Class III lever [24,25] and in vivo clinical measurements, including T-Scan analyses, demonstrating significantly higher bite capacities concentrated in the first molar region [26,27,28,29].

The modeling approach, including boundary conditions and mesh refinement strategies, was developed following the methodological framework established by Chen et al. [17]. While our loading protocol was tailored to our specific clinical configuration, the underlying modeling logic was designed to be consistent with their computational framework.

## 3. Results

Overall evaluation of the models revealed that parafunctional loading conditions consistently generated higher stress values compared to functional loading. This increasing trend was observed under both vertical and oblique loading scenarios. The highest stress values were predominantly localized within the abutment component, whereas the lowest values were recorded in the trabecular bone. Stress accumulations in the cortical bone were significantly higher than those in the trabecular bone, with stress concentrations primarily localized at the implant cervix and the surrounding peri-implant cortical bone regions.

Under vertical functional loading, the maximum stress values were recorded as 222.886 MPa for the implant (Model 17), 468.588 MPa for the abutment (Model 15), and 135.678 MPa for the retaining screw (Model 20). When subjected to parafunctional vertical loading, the peak stress values for these corresponding components increased to 557.215 MPa, 1171.470 MPa, and 339.196 MPa, respectively.

Under oblique functional loading conditions, the maximum stress values were 234.320 MPa for the implant (Model 17), 519.953 MPa for the abutment (Model 20), and 148.676 MPa for the screw (Model 20). Under parafunctional oblique loading scenarios, these maximum stress values escalated to 780.285 MPa for the implant (Model 17), 1731.442 MPa for the abutment (Model 20), and 495.090 MPa for the retaining screw (Model 20).

Regarding the biological and restorative structures under vertical functional loading, the maximum stress values were recorded as 99.288 MPa for the cortical bone (Model 07), 5.000 MPa for the trabecular bone (Model 07), and 412.506 MPa for the superstructure (Model 02). Under vertical parafunctional conditions, the peak stresses reached 248.219 MPa in the cortical bone (Model 07), 12.508 MPa in the trabecular bone (Model 17), and 1039.507 MPa in the superstructure (Model 12).

Under oblique functional loading, the maximum recorded stress values were 62.685 MPa for the cortical bone (Model 17), 2.739 MPa for the trabecular bone (Models 15 and 20), and 212.106 MPa for the superstructure (Model 12). Finally, under oblique parafunctional loading conditions, the highest stress concentrations were determined as 208.223 MPa for the cortical bone (Model 07), 9.121 MPa for the trabecular bone (Model 15), and 706.314 MPa for the superstructure (Model 12). These findings are summarized in Table 4 and Table 5.

The number of implants and the prosthetic design were observed to have a pronounced effect on stress distribution. Models supported by three implants generally yielded lower or more balanced stress distributions across the implant, abutment, screw, and bone tissues compared to two-implant-supported models. This trend was particularly evident in splinted, three-implant-supported designs.

When examining the mechanical effect of the presence and direction of a cantilever on the system, the highest stress values were observed to accumulate at the implant region adjacent to the cantilever. Specifically, models featuring a distal cantilever design exhibited notably higher stress levels compared to mesial cantilever models. A critical trend was identified under parafunctional loads: the maximum stress values on the abutment in models with distal cantilever extensions exceeded 890 MPa, which is the yield strength limit of titanium.

Evaluating splinted and unsplinted restoration designs revealed that splinted models (Models 04, 09, 14, 19) distributed stresses more evenly among the implants in the system, whereas in unsplinted models, stresses reached higher localized levels at specific connection components.

When comparing abutment systems under all loading conditions applied in vertical and oblique directions, it was found that models utilizing Ti-base abutments were subjected to much higher stress levels in the implant, abutment, and screw components compared to models utilizing multi-unit abutments. In Ti-base systems, stress was observed to be localized particularly at the internal connection geometry of the implant and the abutment neck. Under oblique parafunctional (bruxism) loading, it was recorded that the maximum von Mises stresses on the abutment in unsplinted Ti-base designed models also exceeded the 890 MPa yield strength of titanium (creating a risk of plastic deformation). Conversely, multi-unit systems managed to keep the components below this critical threshold by distributing the load over a broader surface.

Regarding the superstructure material, the stress values generated in the superstructure component were found to be lower in models using zirconia-supported feldspathic porcelain compared to monolithic zirconia models. However, the impact of material variation on the stresses in the implant, abutment, screw, cortical bone, and trabecular bone remained limited. Consequently, while the effect of the superstructure material was more pronounced on the stress behavior within the restoration itself, the abutment system, cantilever presence, splinting status, and loading direction were observed to be more dominant regarding the stress on the implant and connection components.

Analyzing stress transmission in the bone tissues, it was determined that the principal stress values generated in the cortical bone were considerably higher than the stress levels in the trabecular bone across all models and loading scenarios. Stresses generally reached their maximum in the cortical bone at the cervical (neck) region of the implant, while rapidly attenuating in the trabecular bone and toward the implant apex.

Comparing physiological and parafunctional (bruxism) loading scenarios, it was found that parafunctional forces exhibited a similar distribution pattern (profile) to functional loads across all models but elevated stress levels to much more critical stress concentrations. In particular, oblique parafunctional forces, compared to vertical forces, were observed to create a much more adverse condition in the biomechanical system by increasing bending moments, leading to high stress foci in the implant neck region, abutment interface, and cortical bone. Under oblique parafunctional loading, the highest abutment and screw stresses were recorded in Model 20, which incorporated a Ti-base, zirconia-supported feldspathic porcelain, and an unsplinted three-implant design. This demonstrates that the direction of loading alters the biomechanical behavior of the system independently of the force magnitude alone.

In all design variations (pontic, cantilever, splinted, unsplinted), the highest stress values were consistently measured in the components placed in the first molar region, where the occlusal force was applied most severely (Figure 6, Figure 7, Figure 8, Figure 9, Figure 10, Figure 11, Figure 12, Figure 13, Figure 14, Figure 15, Figure 16 and Figure 17).

## 4. Discussion

In this study, the biomechanical behaviors of implant number, prosthetic design, abutment system biomaterials, and restorative materials under physiological and parafunctional loading were investigated using FEA.

One of the primary strategies for reducing mechanical stress in multi-unit posterior restorations is increasing the number of supporting implants. In the present study, increasing the number of implants from two to three significantly reduced the stress values in all components (cortical/trabecular bone, implant body, abutment, screw, and superstructure) under both axial and oblique loading conditions. These findings are consistent with previous studies suggesting that an increased number of supporting implants expands the load-bearing area and reduces the magnitude of force transmitted to each unit [30,31]. However, an increase in the number of implants should not be viewed as an absolute clinical advantage. Indeed, a long-term clinical study demonstrated that, contrary to laboratory findings, two-implant-supported bridge designs may exhibit higher survival rates than three-implant restorations [32]. Consequently, the biomechanical benefits of increasing implant number must be weighed against clinical requirements, including hygiene maintenance and the achievement of a passive fit.

Furthermore, splinted designs were found to distribute stresses more homogeneously among components compared to independent (unsplinted) designs. It has been confirmed that splinting restorations, especially in scenarios of reduced bone support or excessive occlusal loading, prevents focal stress accumulations and protects the system against bending moments [33,34].

Among the prosthetic designs configured in the mandibular posterior region, the highest stress accumulations were observed in models with distal cantilevers (Models 02, 07, 12, 17). Cantilever extensions create a leverage effect in the system, reflecting stress directly onto the implant adjacent to the extension and the connection components [35,36]. The most critical picture in stress distribution was observed to progress in the order of distal cantilever > mesial cantilever > pontic design [6]. In cases where the use of a cantilever is mandatory, the present results further demonstrate that mesial extensions are biomechanically much safer than distal extensions due to their anatomical and occlusal advantages [7]. The most critical finding of the present study was observed in parafunctional (bruxism) loading scenarios. Because all materials in the present FEA were modeled as linear elastic, the analysis cannot quantitatively capture post-yield plastic behavior. Therefore, maximum principal stress values on the abutment exceeding the 890 MPa yield strength of titanium should be interpreted as a strong indicator of plastic-deformation risk rather than a direct prediction of actual deformation. This risk profile mathematically explains the high incidence of abutment fractures and screw loosening associated with posterior cantilever restorations in clinical practice [37].

The retention concept and abutment geometry play a decisive role in damping occlusal forces. In this study, Ti-base systems were found to generate higher stress values in the implant, screw, and abutment components compared to multi-unit systems. The interlocking geometry of Ti-base abutments, which integrates into the internal structure of the implant, creates a “wedge effect” under applied forces, localizing stress in a narrow area [38]. In contrast, multi-part multi-unit systems, which elevate the restorative platform to the supragingival level, were observed to distribute forces more evenly, thereby protecting the implant and bone interface [39,40]. However, it should be considered that Ti-base systems may provide advantages in esthetic zones, and their generation of higher stress does not mean absolute clinical failure.

When evaluating the stresses within the superstructure itself, higher internal stress concentrations were detected in monolithic zirconia restorations due to their high elastic modulus. However, based on the literature, the high fracture toughness of monolithic zirconia allows it to tolerate these internal stresses. Furthermore, the single-piece monolithic structure completely eliminates veneer chipping, a frequent complication of layered ceramic restorations [41,42]. This literature-supported interpretation suggests that monolithic zirconia is a highly predictable biomaterial alternative in the rehabilitation of the posterior region, where excessive forces are prevalent.

One of the strongest methodological aspects of the present study is the configuration of the occlusal loading protocol to simulate physiological and parafunctional masticatory dynamics with high precision. While many FEA studies in the literature adopt simplified, homogeneous loading scenarios where total occlusal force is distributed equally—a strategy that often oversimplifies the biomechanical environment—this study employs a ‘proportional loading’ strategy. By distributing forces to the first molar, second premolar, and first premolar at ratios of 50%, 30%, and 20% respectively, we aligned our model with the well-established Class III leverage system of the mandible, where force magnitude peaks in the posterior region [25,27,29]. This approach minimizes the risk of underestimating stress concentrations on posterior implants and provides a more clinically relevant representation of the actual load transfer mechanism.

The parafunctional (bruxism) scenarios tested under this realistic loading strategy clearly revealed the destructive effect of not only the magnitude but also the direction of occlusal forces on the system. In the literature, bruxism is known to increase normal masticatory forces by 4 to 7 times and shift their direction from axial to oblique [22,43]. Similarly, in our study, oblique parafunctional forces (total 500 N) applied at a 30-degree angle in the buccolingual direction caused much more aggressive stress accumulations in all configurations compared to vertical functional forces (total 400 N).

The findings of this study are in accordance with previous studies highlighting the biomechanical impact of loading angles and patient-related factors. Oblique loading generates significantly higher stress levels compared to vertical loading, potentially exceeding safety limits, particularly in narrow-diameter connections [44]. Furthermore, oblique forces have been shown to increase equivalent stress by 33% and maximum principal stress by 60% within the restoration system [22]. The destructive potential of parafunctional activities, such as bruxism, is clinically critical; existing evidence confirms that connection complexes are the weakest link in the system, with bruxism increasing the risk of screw loosening by 3.3-fold, screw fracture by 4.9-fold, and ceramic fractures by 5.6-fold [45]. However, other studies offer a broader clinical perspective by suggesting that bruxism should not be considered an absolute contraindication, but rather a risk factor that requires meticulous management [11,46]. Within this risk management framework, it is advocated that cantilevers be eliminated, the occlusal table reduced, and cuspal inclinations flattened to tolerate bruxism [4,47]. Furthermore, a protective treatment approach using canine-protected occlusion, as opposed to group function, has been demonstrated to radically reduce stress transmitted to the bone and implant [48].

It is crucial to note that all materials in this study were modeled as linear elastic. Consequently, the analysis cannot quantitatively capture plastic behavior once the yield strength of a material is exceeded. In this context, maximum principal and von Mises stress values exceeding the 890 MPa yield strength of the Titanium Alloy (Grade 5, Ti-6Al-4V) must be interpreted strictly as indicators of a high risk for plastic deformation, rather than actual predictions of physical deformation or immediate clinical failure. These values represent biomechanical risk trends that arise from the specific modeling assumptions of this in silico study.

A notable finding of the study was that under simulated parafunctional oblique loading, stress values exceeded the yield strength of the titanium alloy (890 MPa) in designs with distal cantilevers and in unsplinted models using Ti-base abutments. Because all materials in this study were modeled as linear elastic, these elevated stress values cannot quantitatively capture actual physical deformation; rather, they serve as indicators of a high risk for plastic deformation. These outcomes represent biomechanical risk trends that align with the underlying causes of mechanical complications frequently encountered in bruxist individuals in clinical practice, such as screw loosening, screw fracture, and ceramic chipping [45]. Consequently, these theoretical stress concentrations suggest that, although bruxism is not a direct contraindication for implant therapy, it acts as a major biomechanical risk factor that necessitates protective restorative strategies, such as appropriate abutment selection (e.g., the use of multi-unit abutments), splinting, and the elimination of cantilevers.

When examining the transmission of forces to the implant–bone interface, it was observed that the highest stress concentrations were localized in the cortical bone at the cervical region of the implant in every scenario and decreased apically. The maximum and minimum principal stress values generated in the cortical bone were significantly higher than the values in the trabecular bone. This finding is in full agreement with previous research reporting that trabecular bone, which has a low elastic modulus, shows weak resistance to applied loads, and that the majority of occlusal loads are absorbed by the crestal cortical bone [49,50]. This intense stress accumulation in the cortical bone explains the biomechanical cause of marginal bone resorption and demonstrates once again how critical the careful management of occlusal loads is for the long-term survival of the implant.

Although our specific loading parameters differ from those in the study by Chen et al. [17] due to our unique clinical scenario, the resulting stress distribution patterns exhibit similar biomechanical trends. This comparison serves purely as a qualitative consistency check rather than a quantitative validation. The observations in our simulations suggest that our findings align with established computational behaviors in mandibular posterior implant cases [17]; however, the current findings must be interpreted strictly as relative biomechanical risk trends.

Although the data obtained from our study strongly align with the current literature, the results should be interpreted within the framework of certain methodological limitations inherent to in silico analyses. Most notably, the finite element model was not independently validated against experimental or physical benchmark data. The complex kinematics of the human masticatory system and the dynamic effect of muscle forces cannot be perfectly replicated in a computational environment. The limitations of the study include the assumptions in the FEA simulations that the materials are homogeneous and linear elastic, and that the bone-implant interface is 100% osseointegrated. The findings obtained should be evaluated as a guide shedding light on the biomechanical risk profiles of different prosthetic designs, strictly representing relative biomechanical risk trends, rather than as absolute clinical failure rates.

While this FEA study provides crucial comparative biomechanical insights for implant configurations under bruxism, it is inherently limited by its computational nature. Assumptions such as complete osseointegration and static loading conditions do not fully mirror the dynamic biological environment. Therefore, the findings should be interpreted as a qualitative baseline. Future in vitro mechanical fatigue testing and long-term clinical trials are necessary to experimentally validate these theoretical outcomes.

## 5. Conclusions

Within the limitations of this in silico study, the following biomechanical trends were identified:

1. Increasing the number of implants from two to three and opting for splinted designs substantially reduced stresses on the entire prosthetic system and bone tissue.
2. Among the prosthetic designs, the highest stress accumulations were observed in distal cantilever configurations. Pontic designs should be prioritized; in obligatory cases, the mesial cantilever presents a lower biomechanical risk.
3. Ti-base abutment systems transmitted occlusal loads more locally, causing noticeably higher stresses in the implant and screw complex compared to multi-unit systems.
4. Under vertical and oblique parafunctional (bruxism) loading, models with distal cantilevers and unsplinted designs using Ti-base abutments exhibited abutment stresses exceeding the yield strength of titanium (890 MPa). These specific combinations indicate a high risk of plastic deformation and should be avoided in patients with bruxism.
5. The selection of the restorative superstructure material has a negligible biomechanical impact on the stress profile transmitted to the implant components and surrounding bone.

## Figures and Tables

**Figure 6:**
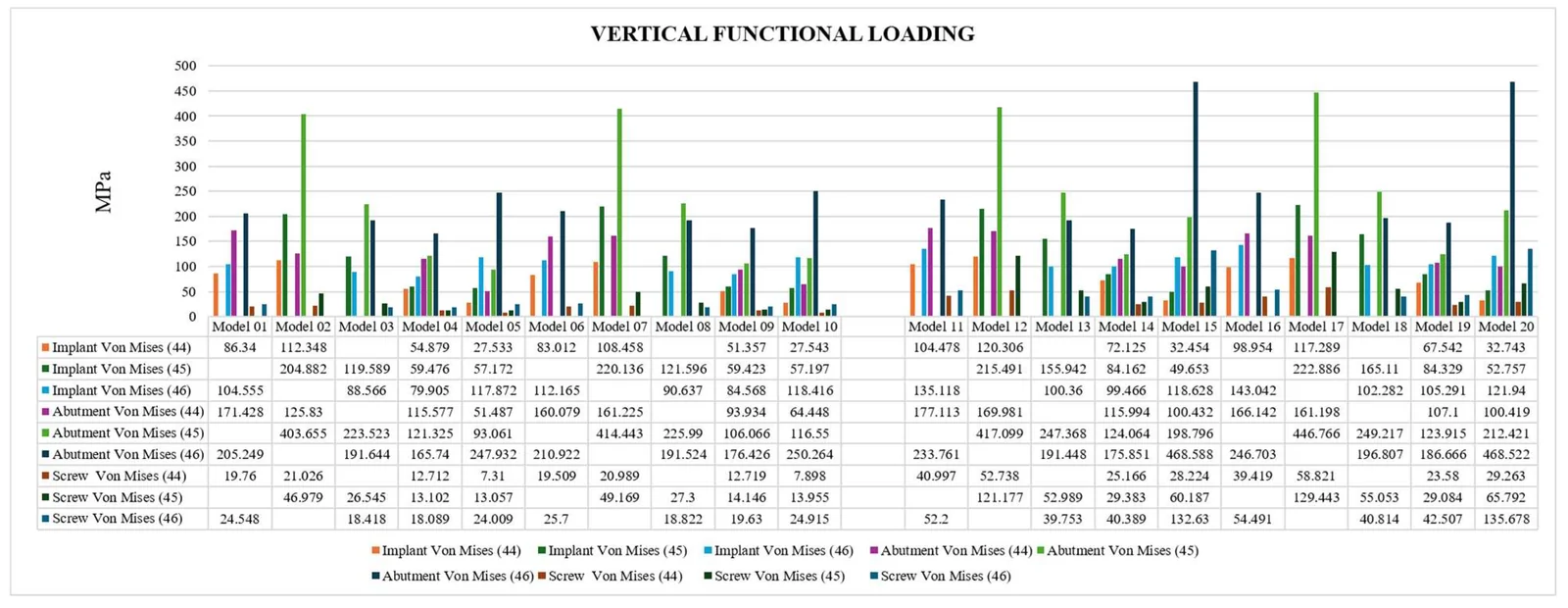
Von Mises stress values in the implants, abutments, and screws of Models (01–20) under vertical functional loading.

**Figure 7:**
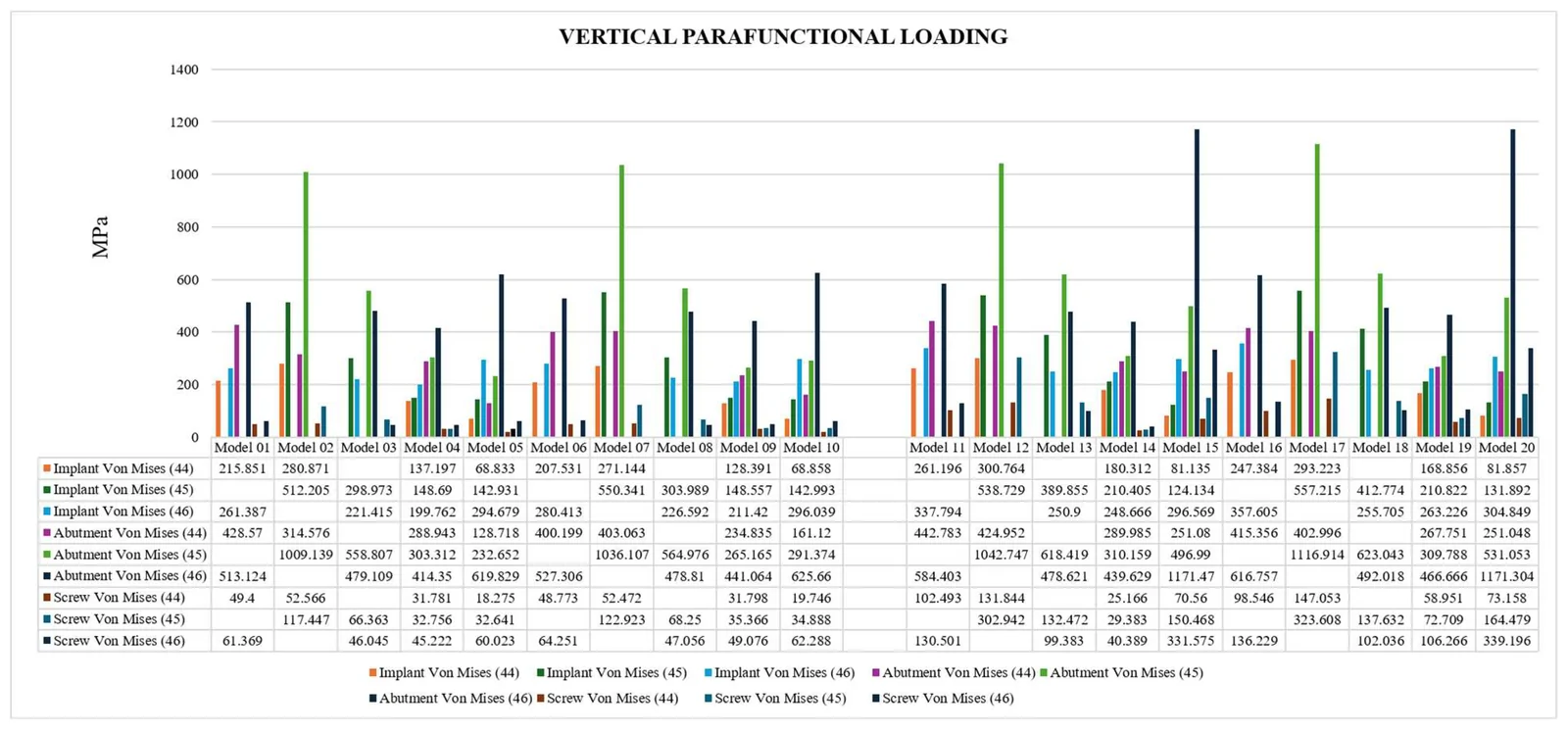
Von Mises stress values in the implants, abutments, and screws of Models (01–20) under vertical parafunctional loading. Values exceeding 890 MPa (Ti-6Al-4V yield strength) represent a high risk of plastic deformation.

**Figure 8:**
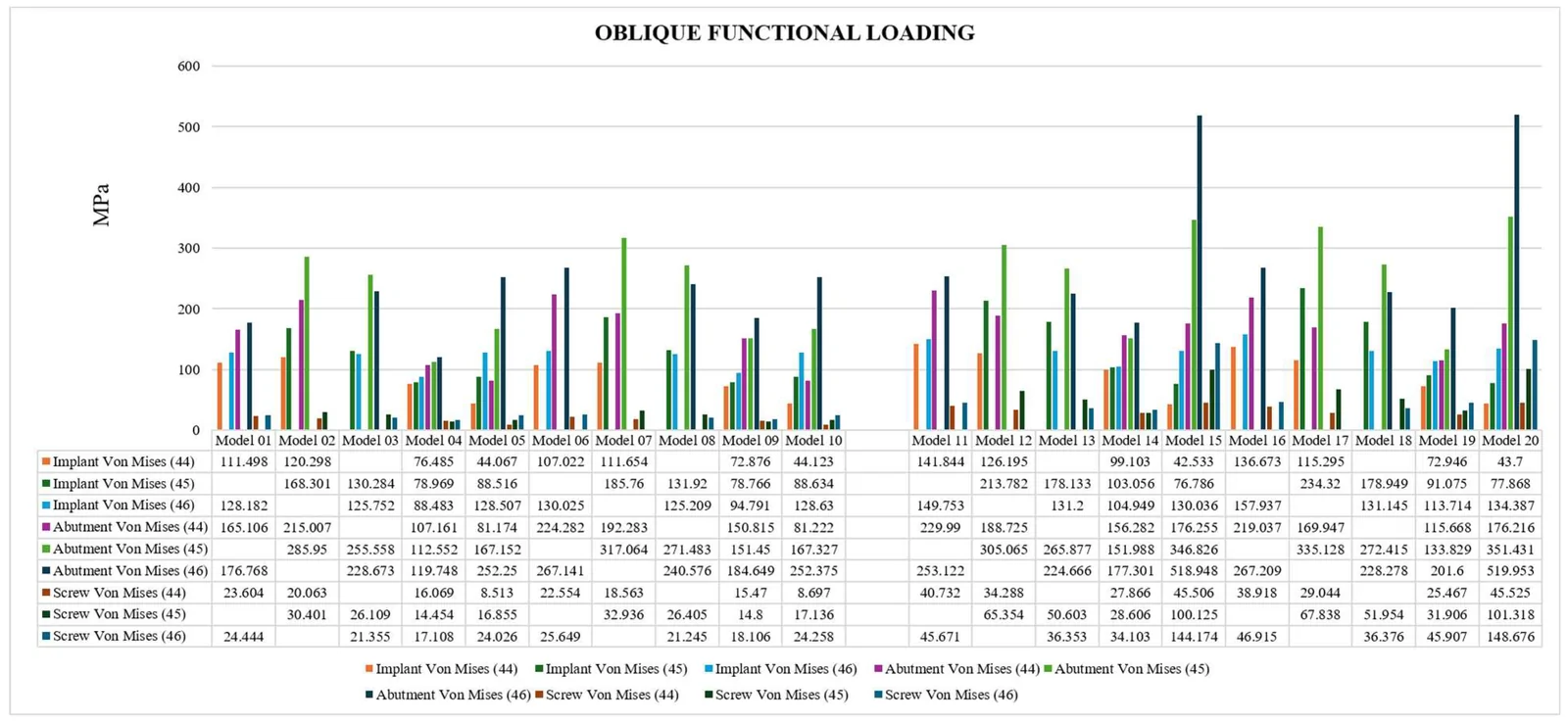
Von Mises stress values in the implants, abutments, and screws of Models (01–20) under oblique functional loading.

**Figure 9:**
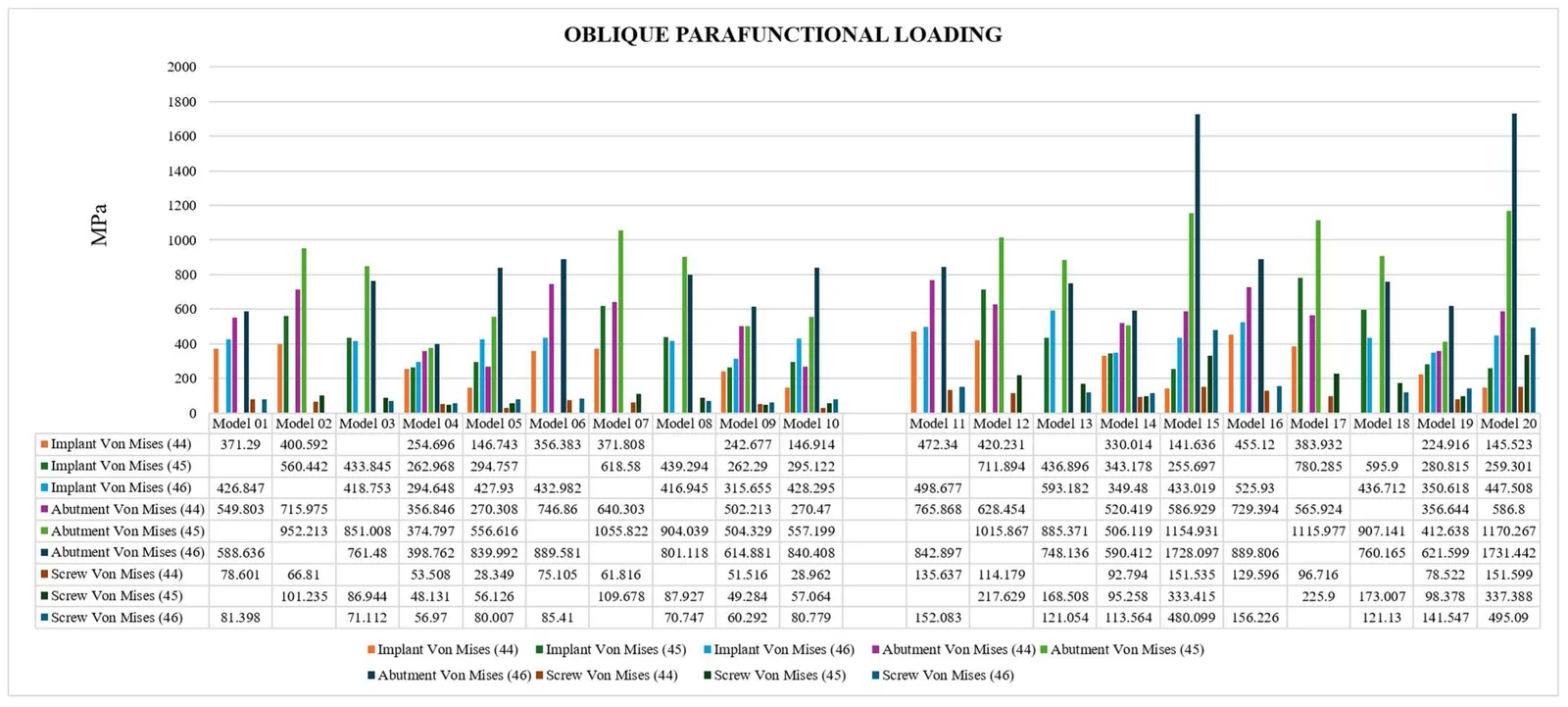
Von Mises stress values in the implants, abutments, and screws of Models (01–20) under oblique parafunctional loading. Values exceeding 890 MPa (Ti-6Al-4V yield strength) represent a high risk of plastic deformation.

**Figure 10:**
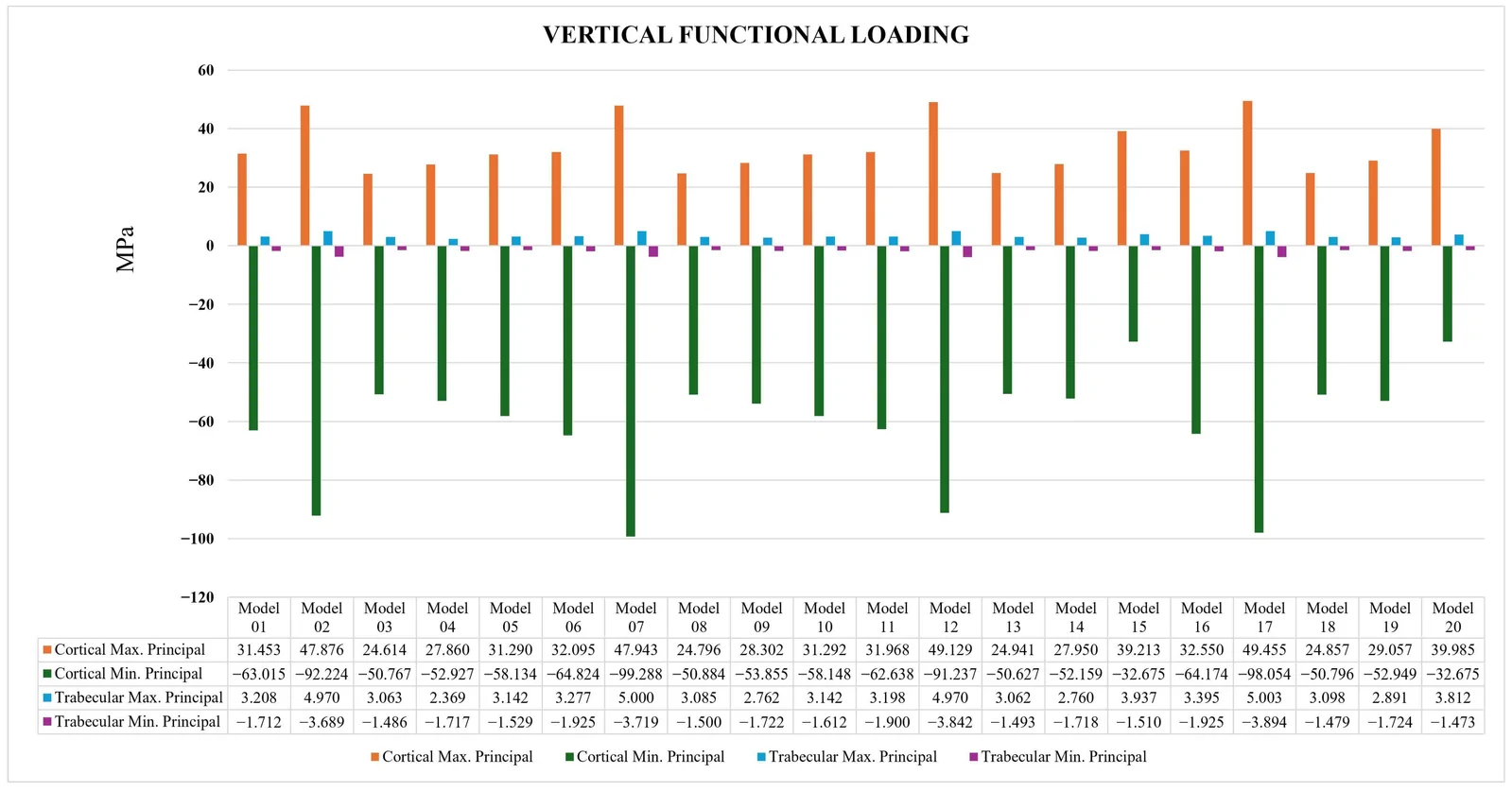
Maximum and minimum principal stress values in the cortical and trabecular bones of Models (01–20) under vertical functional loading.

**Figure 11:**
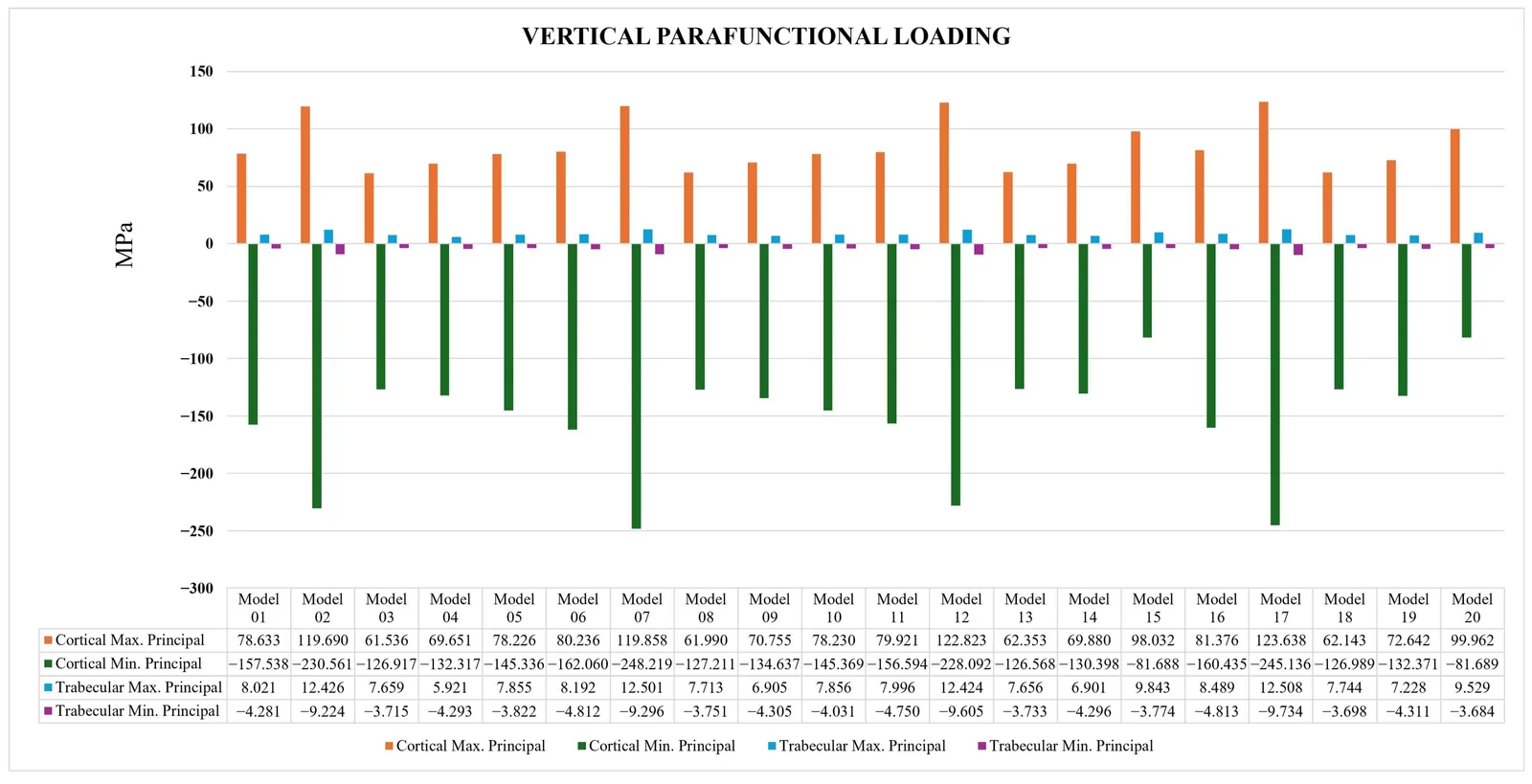
Maximum and minimum principal stress values in the cortical and trabecular bones of Models (01–20) under vertical parafunctional loading.

**Figure 12:**
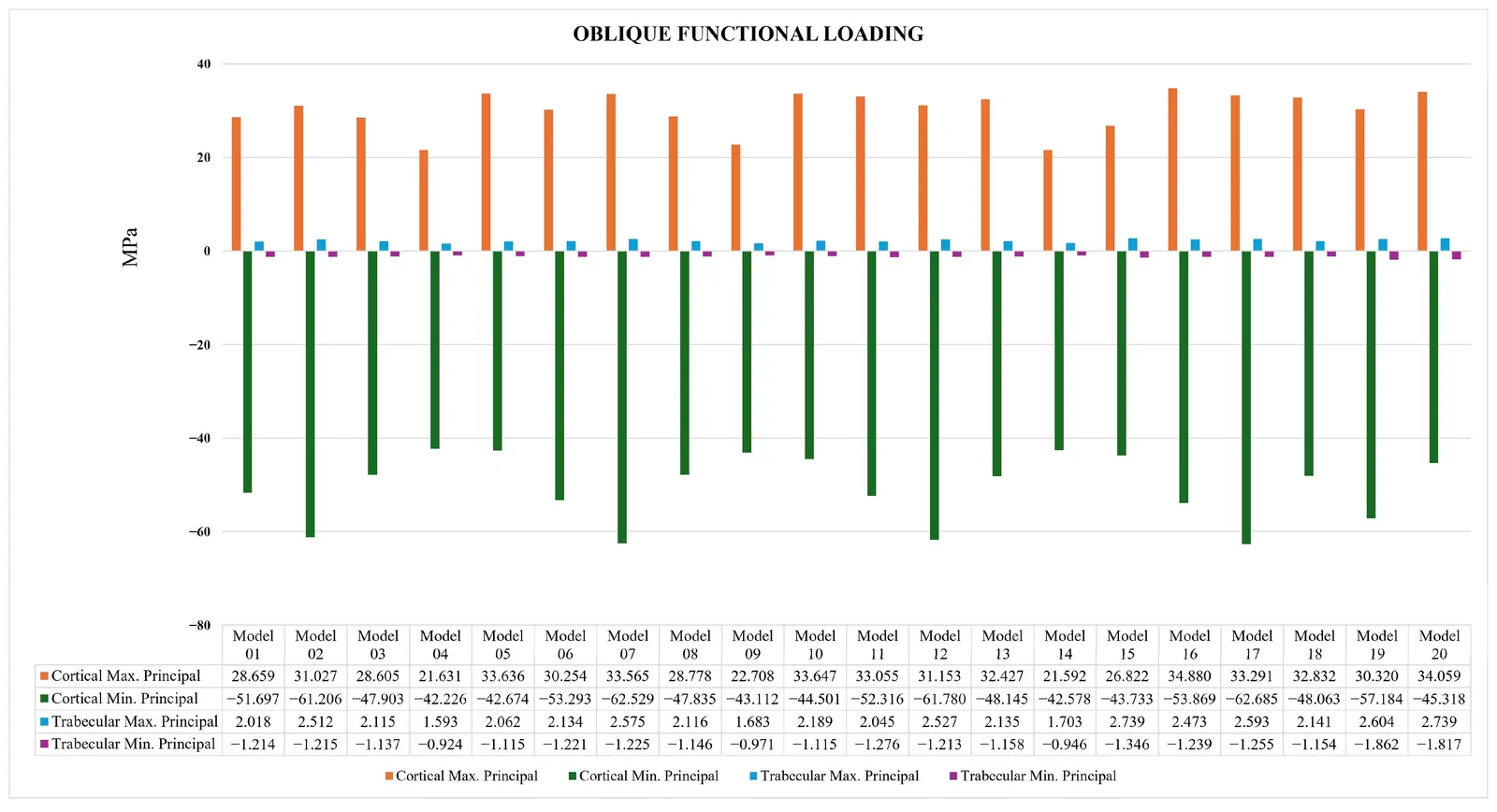
Maximum and minimum principal stress values in the cortical and trabecular bones of Models (01–20) under oblique functional loading.

**Figure 13:**
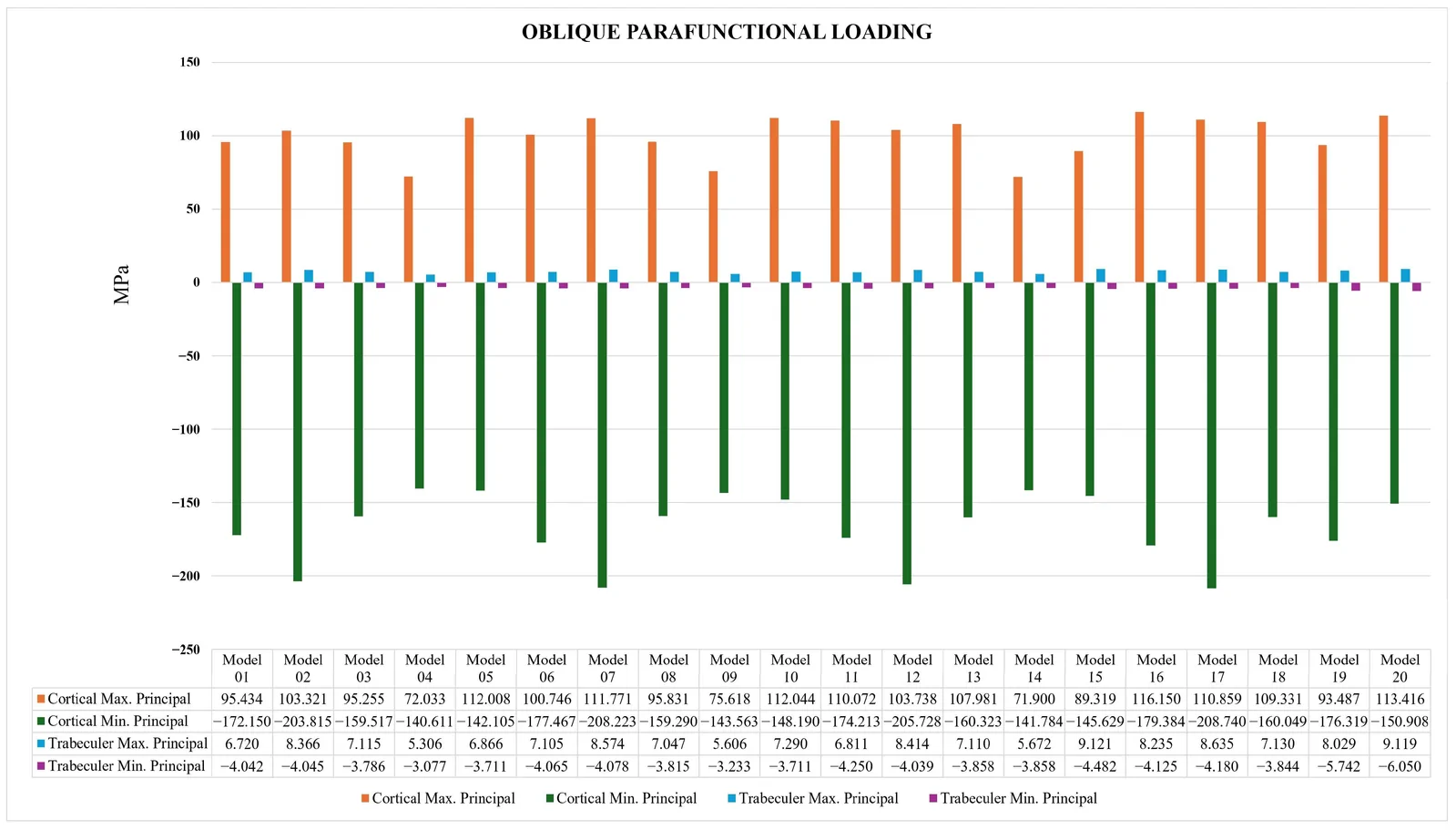
Maximum and minimum principal stress values in the cortical and trabecular bones of Models (01–20) under oblique parafunctional loading.

**Figure 14:**
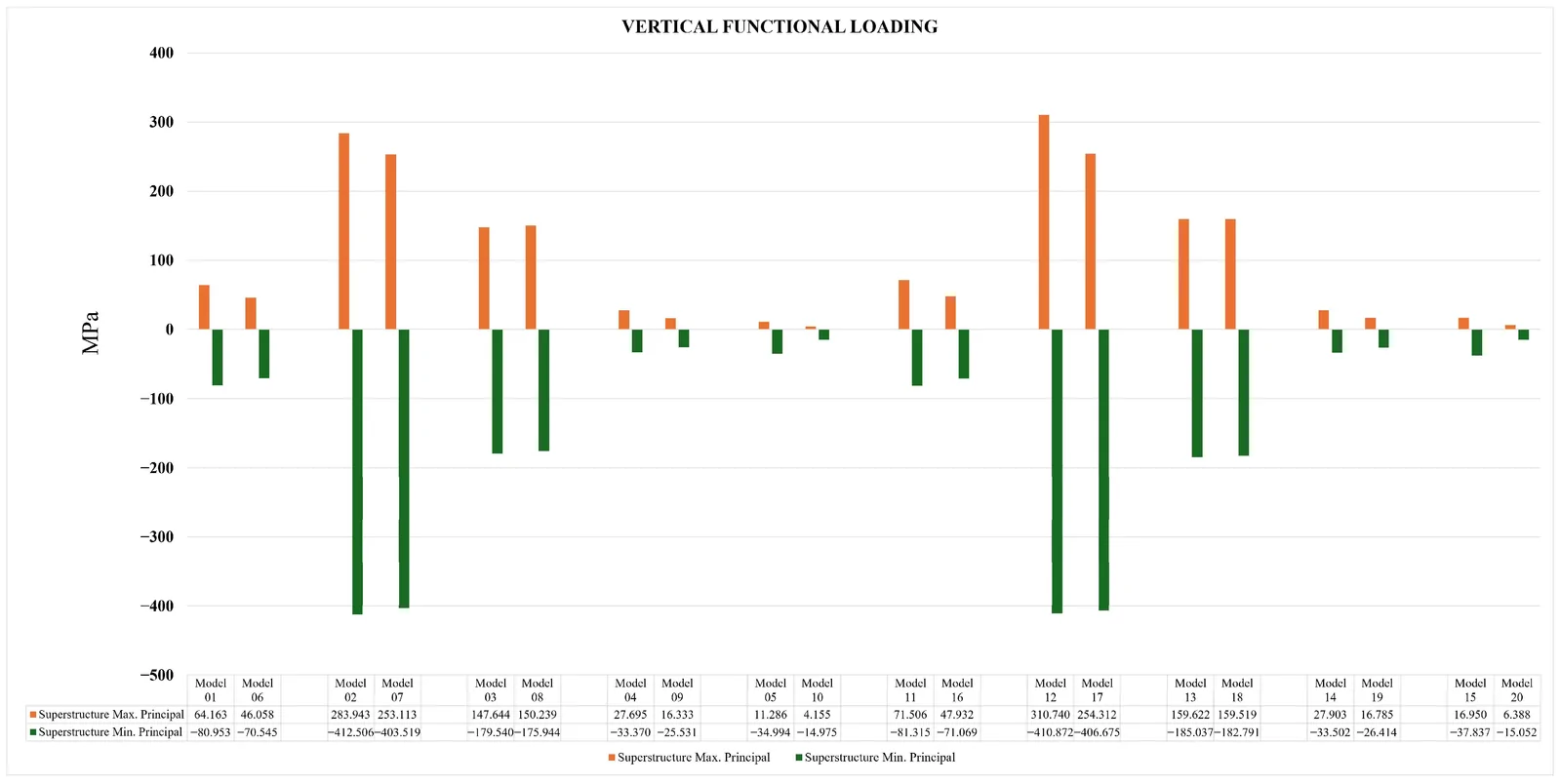
Maximum and minimum principal stress values in the superstructures of Models (01, 06, 02, 07, 03, 08, 04, 09, 05, 10, 11, 16, 12, 17, 13, 18, 14, 19, 15, 20) under vertical functional loading.

**Figure 15:**
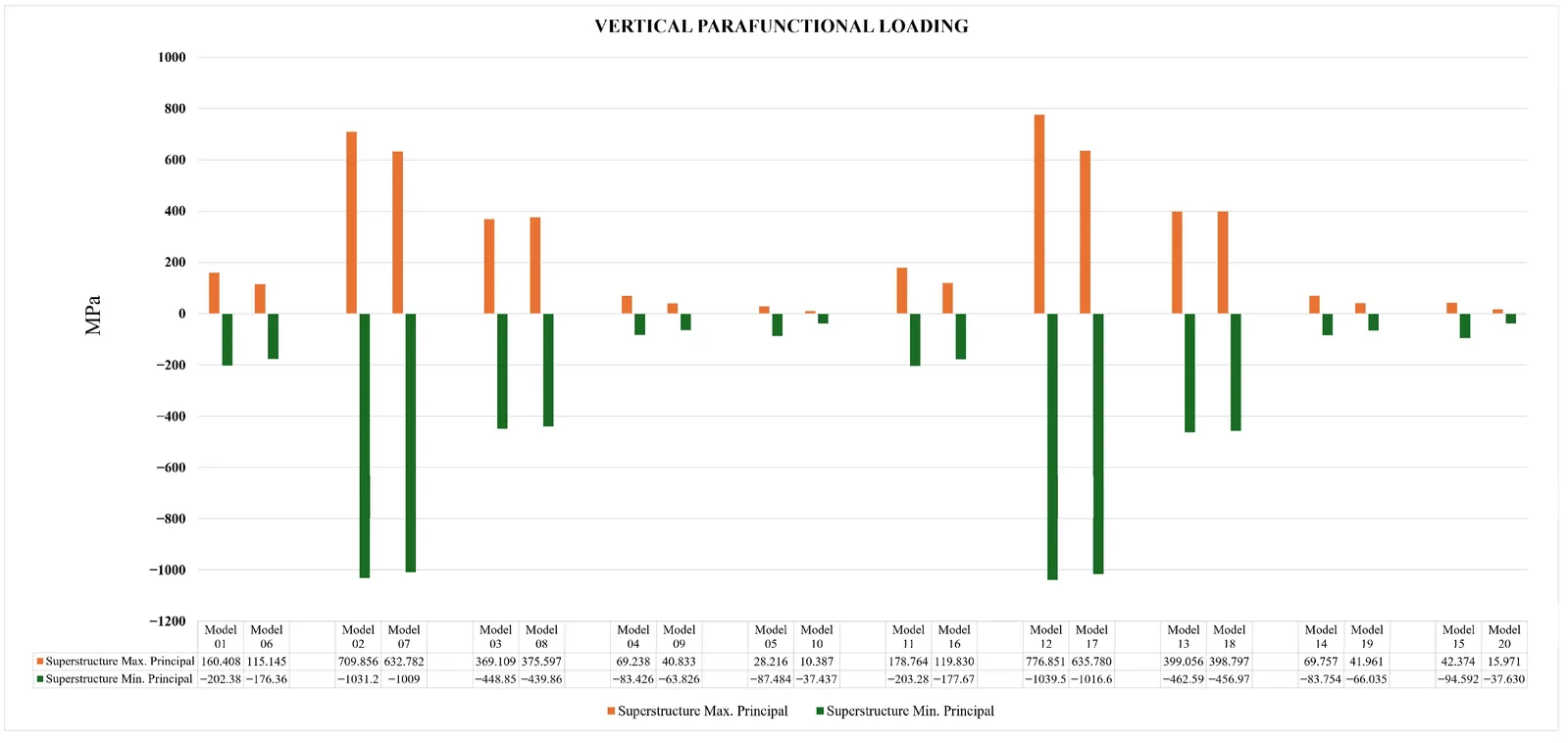
Maximum and minimum principal stress values in the superstructures of Models (01, 06, 02, 07, 03, 08, 04, 09, 05, 10, 11, 16, 12, 17, 13, 18, 14, 19, 15, 20) under vertical parafunctional loading.

**Figure 16:**
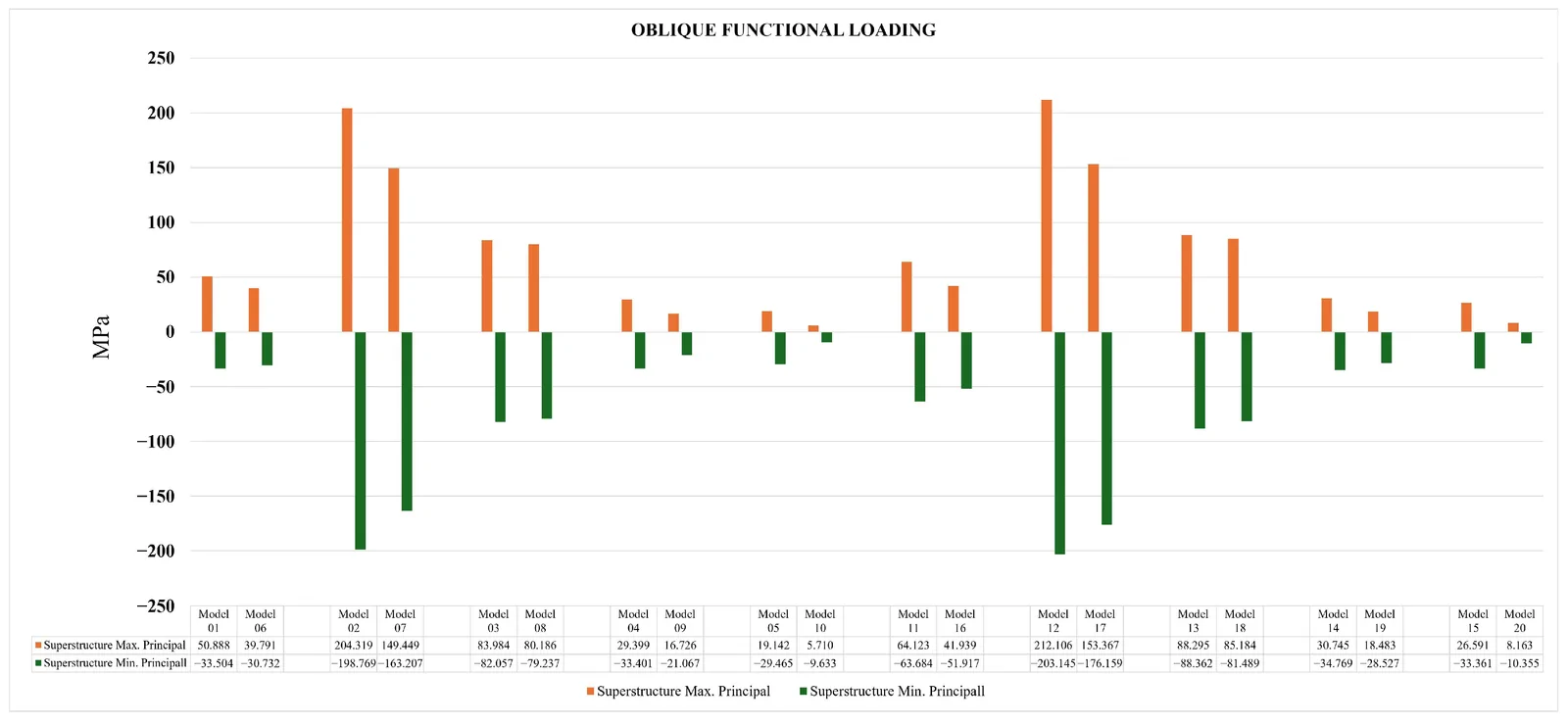
Maximum and minimum principal stress values in the superstructures of Models (01, 06, 02, 07, 03, 08, 04, 09, 05, 10, 11, 16, 12, 17, 13, 18, 14, 19, 15, 20) under oblique functional loading.

**Figure 17:**
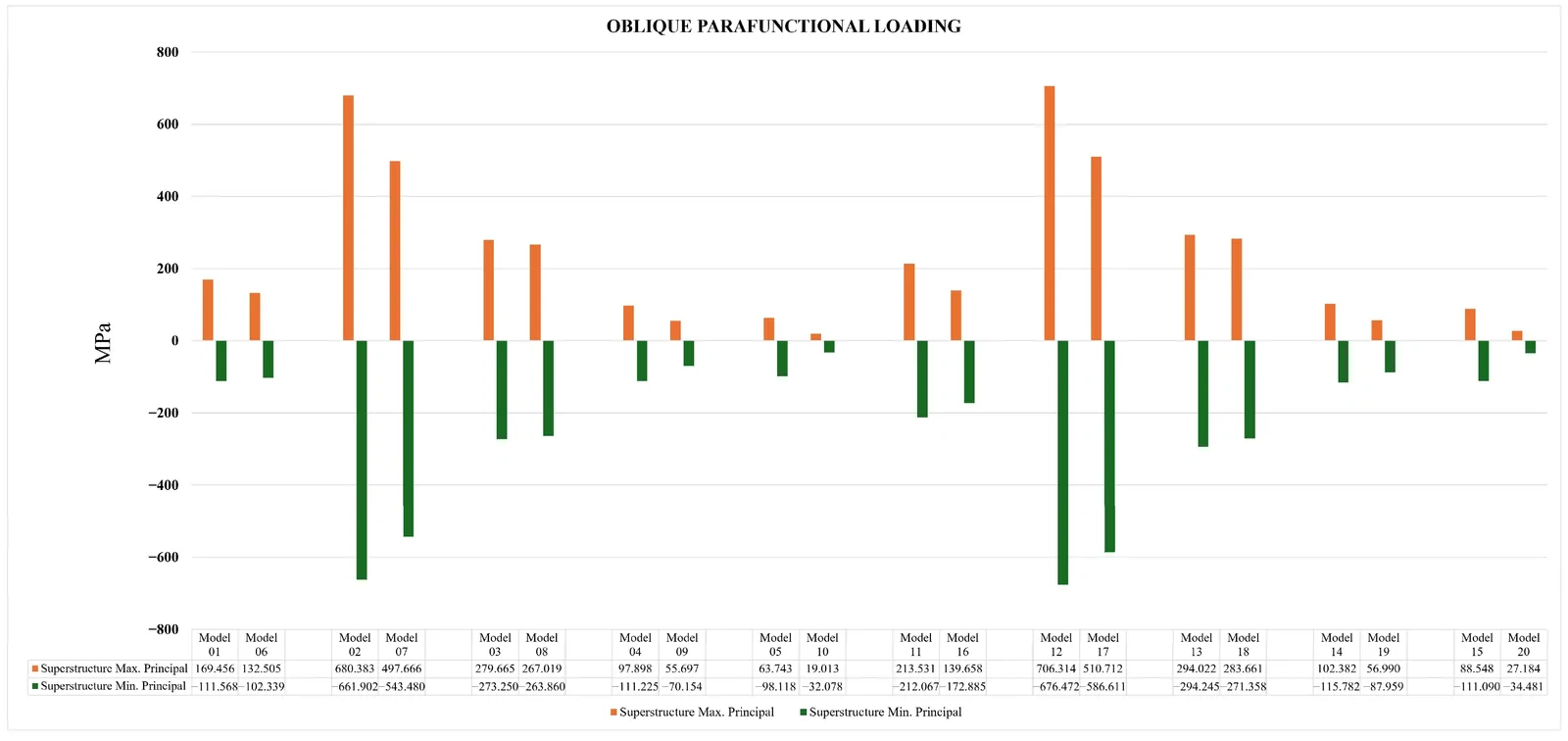
Maximum and minimum principal stress values in the superstructures of Models (01, 06, 02, 07, 03, 08, 04, 09, 05, 10, 11, 16, 12, 17, 13, 18, 14, 19, 15, 20) under oblique parafunctional loading.

**Table 1:** Model groups and prosthetic variables evaluated in the study.

Model	Abutment System	Superstructure Material	Prosthetic Design
Model 01	Multi-unit	Monolithic zirconia	Pontic (2 implants)
Model 02	Multi-unit	Monolithic zirconia	Distal cantilever (2 implants)
Model 03	Multi-unit	Monolithic zirconia	Mesial cantilever (2 implants)
Model 04	Multi-unit	Monolithic zirconia	Splinted (3 implants)
Model 05	Multi-unit	Monolithic zirconia	Non-splinted (3 implants)
Model 06	Multi-unit	Zirconia framework with porcelain	Pontic (2 implants)
Model 07	Multi-unit	Zirconia framework with porcelain	Distal cantilever (2 implants)
Model 08	Multi-unit	Zirconia framework with porcelain	Mesial cantilever (2 implants)
Model 09	Multi-unit	Zirconia framework with porcelain	Splinted (3 implants)
Model 10	Multi-unit	Zirconia framework with porcelain	Non-splinted (3 implants)
Model 11	Ti-base	Monolithic zirconia	Pontic (2 implants)
Model 12	Ti-base	Monolithic zirconia	Distal cantilever (2 implants)
Model 13	Ti-base	Monolithic zirconia	Mesial cantilever (2 implants)
Model 14	Ti-base	Monolithic zirconia	Splinted (3 implants)
Model 15	Ti-base	Monolithic zirconia	Non-splinted (3 implants)
Model 16	Ti-base	Zirconia framework with porcelain	Pontic (2 implants)
Model 17	Ti-base	Zirconia framework with porcelain	Distal cantilever (2 implants)
Model 18	Ti-base	Zirconia framework with porcelain	Mesial cantilever (2 implants)
Model 19	Ti-base	Zirconia framework with porcelain	Splinted (3 implants)
Model 20	Ti-base	Zirconia framework with porcelain	Non-splinted (3 implants)

**Table 2:** Mechanical Properties Ascribed to Materials in the Model.

Material	Elastic Modulus (MPa)	Poisson’s Ratio	References
Monolithic Zirconia (3Y-TZP)	210,000	0.30	[16]
Titanium Alloy (Grade 5, Ti-6Al-4V)	110,000	0.35	[17]
Feldspathic Porcelain	82,800	0.35	[18]
Dual-cure Resin Cement	18,600	0.28	[19]
Cortical Bone	13,700	0.30	[17]
Trabecular Bone	1370	0.30	[17]

**Table 3:** Static loading scenarios applied in the study and force distributions by region.

Scenario Type	Force Direction	1st Premolar	2nd Premolar	1st Molar	Total Force
1. Functional	Vertical (Axial)	80 N (2 points)	120 N (2 points)	200 N (4 points)	400 N
2. Parafunctional	Vertical (Axial)	200 N (2 points)	300 N (2 points)	500 N (4 points)	1000 N
3. Functional	Oblique (30°)	30 N (2 points)	45 N (2 points)	75 N (4 points)	150 N
4. Parafunctional	Oblique (30°)	100 N (2 points)	150 N (2 points)	250 N (4 points)	500 N

**Table 4:** Models exhibiting the maximum stresses generated in the components according to loading conditions and their values (MPa).

Component	Vert. Functional (MPa)	Model Configuration	Vert. Parafunctional (MPa)	Model Configuration	Oblique Functional (MPa)	Model Configuration	Oblique Parafunctional (MPa)	Model Configuration
Implant	222.886	M17 (TB, Zr-P, Distal cantilever)	557.215	M17 (TB, Zr-P, Distal cantilever)	234.320	M17 (TB, Zr-P, Distal cantilever)	780.285	M17 (TB, Zr-P, Distal cantilever)
Abutment	468.588	M15 (TB, MZ, Unsplinted 3 imp)	1171.470	M15 (TB, MZ, Unsplinted 3 imp)	519.953	M20 (TB, MZ, Unsplinted 3 imp)	1731.442	M20 (TB, MZ, Unsplinted 3 imp)
Screw	135.678	M20 (TB, Zr-P, Unsplinted 3 imp)	339.196	M20 (TB, Zr-P, Unsplinted 3 imp)	148.676	M20 (TB, Zr-P, Unsplinted 3 imp)	495.090	M20 (TB, Zr-P, Unsplinted 3 imp)
Cortical Bone	99.288	M07 (MU, Zr-P, Distal cantilever)	248.219	M07 (MU, Zr-P, Distal cantilever)	62.685	M17 (TB, Zr-P, Distal cantilever)	208.223	M07 (MU, Zr-P, Distal cantilever)
Trabecular Bone	5.000	M07 (MU, Zr-P, Distal cantilever)	12.508	M17 (TB, Zr-P, Distal cantilever)	2.739	M15 & M20 (Unsplinted 3 imp)	9.121	M15 (TB, MZ, Unsplinted 3 imp)
Superstructure	412.506	M02 (MU, MZ, Distal cantilever)	1039.507	M12 (TB, MZ, Distal cantilever)	212.106	M12 (TB, MZ, Distal cantilever)	706.314	M12 (TB, MZ, Distal cantilever)

Mpa: Megapascal, TB: Titanium Base, MU: Multi-unit, MZ: Monolithic Zirconia, Zr-P: Porcelain-veneered Zirconia.

**Table 5:** Summary of the biomechanical trends based on evaluated prosthetic variables.

Prosthetic Variable	Biomechanical Trend	Effect/Observation
Implant Number	3 implants > 2 implants	Lower stress distribution
Cantilever Design	Pontic > Mesial Cantilever > Distal Cantilever	Safest to riskiest
Splinting	Splinted > Unsplinted	Better load distribution
Abutment System	Multi-unit > Ti-base	Lower localized stress in the connection
Superstructure Material	Monolithic Zirconia ≈ Porcelain-veneered	Negligible effect on implant/bone stress

## Data Availability

The data presented in this study are available on request from the corresponding author.

## Abbreviations

The following abbreviations are used in this manuscript:

FEA	Finite Element Analysis
CT	Computed Tomography
MPa	Megapascal
Ti	Titanium
Ti-base	Titanium Base
MU	Multi-unit
MZ	Monolithic Zirconia
Zr-P	Porcelain-veneered Zirconia
Pmax	Maximum Principal Stress
N	Newton
